# Development of complemented comprehensive networks for rapid screening of repurposable drugs applicable to new emerging disease outbreaks

**DOI:** 10.1186/s12967-023-04223-2

**Published:** 2023-06-26

**Authors:** Yonghyun Nam, Anastasia Lucas, Jae-Seung Yun, Seung Mi Lee, Ji Won Park, Ziqi Chen, Brian Lee, Xia Ning, Li Shen, Anurag Verma, Dokyoon Kim

**Affiliations:** 1 Department of Biostatistics, Epidemiology & Informatics, The Perelman School of Medicine, University of Pennsylvania, B304 Richards Building, 3700 Hamilton Walk, Philadelphia, PA 19104-6116 USA; 2 Genomics and Computational Biology Graduate Group, Perelman School of Medicine, University of Pennsylvania, Philadelphia, USA; 3 Division of Endocrinology and Metabolism, Department of Internal Medicine, St. Vincent’s Hospital, College of Medicine, The Catholic University of Korea, Seoul, South Korea; 4 Department of Obstetrics and Gynecology, Seoul National University College of Medicine, Seoul, South Korea; 5 Department of Surgery, Seoul National University College of Medicine, Seoul, South Korea; 6 Computer Science and Engineering Department, College of Engineering, The Ohio State University, Columbus, USA; 7 Department of Medicine, Perelman School of Medicine, University of Pennsylvania, Philadelphia, USA; 8 Biomedical Informatics Department, College of Medicine, The Ohio State University, Columbus, USA; 9 Translational Data Analytics Institute, The Ohio State University, Columbus, USA; 10 Institute for Biomedical Informatics, University of Pennsylvania, Philadelphia, USA

**Keywords:** Drug repurposing, Network medicine, Graph-based semi-supervised learning, COVID-19

## Abstract

**Background:**

Computational drug repurposing is crucial for identifying candidate therapeutic medications to address the urgent need for developing treatments for newly emerging infectious diseases. The recent COVID-19 pandemic has taught us the importance of rapidly discovering candidate drugs and providing them to medical and pharmaceutical experts for further investigation. Network-based approaches can provide repurposable drugs quickly by leveraging comprehensive relationships among biological components. However, in a case of newly emerging disease, applying a repurposing methods with only pre-existing knowledge networks may prove inadequate due to the insufficiency of information flow caused by the novel nature of the disease.

**Methods:**

We proposed a network-based complementary linkage method for drug repurposing to solve the lack of incoming new disease-specific information in knowledge networks. We simulate our method under the controlled repurposing scenario that we faced in the early stage of the COVID-19 pandemic. First, the disease-gene-drug multi-layered network was constructed as the backbone network by fusing comprehensive knowledge database. Then, complementary information for COVID-19, containing data on 18 comorbid diseases and 17 relevant proteins, was collected from publications or preprint servers as of May 2020. We estimated connections between the novel COVID-19 node and the backbone network to construct a complemented network. Network-based drug scoring for COVID-19 was performed by applying graph-based semi-supervised learning, and the resulting scores were used to validate prioritized drugs for population-scale electronic health records-based medication analyses.

**Results:**

The backbone networks consisted of 591 diseases, 26,681 proteins, and 2,173 drug nodes based on pre-pandemic knowledge. After incorporating the 35 entities comprised of complemented information into the backbone network, drug scoring screened top 30 potential repurposable drugs for COVID-19. The prioritized drugs were subsequently analyzed in electronic health records obtained from patients in the Penn Medicine COVID-19 Registry as of October 2021 and 8 of these were found to be statistically associated with a COVID-19 phenotype.

**Conclusion:**

We found that 8 of the 30 drugs identified by graph-based scoring on complemented networks as potential candidates for COVID-19 repurposing were additionally supported by real-world patient data in follow-up analyses. These results show that our network-based complementary linkage method and drug scoring algorithm are promising strategies for identifying candidate repurposable drugs when new emerging disease outbreaks.

**Supplementary Information:**

The online version contains supplementary material available at 10.1186/s12967-023-04223-2.

## Introduction

During the recent COVID-19 pandemic caused by the severe acute respiratory syndrome coronavirus-2 (SARS-CoV-2), many researchers and pharmaceutical companies have conducted numerous studies for developing treatments and vaccines. In particular, many researchers attempt to repurpose known drugs to treat patients with SARS-CoV-2 infection because drug repurposing is cheaper and quicker than conventional drug discovery. Drug repurposing aims to find new indicators in already-approved drugs that could be used for other diseases [1–3]. Several drugs have been successfully repositioned for COVID-19, such as remdesivir (initially developed to treat the Ebola virus) and dexamethasone (used in conditions for anti-inflammatory and immunosuppressant effects) [4–6]. Although the global COVID-19 pandemic is gradually shifting to an endemic stage thanks to the development of vaccines and treatments, the spread of coronavirus is still ongoing as of September 2022. One of the lessons we have learned from the recent pandemic is that it is important to rapidly discover a list of candidate drugs and provide it to experts in the medical or pharmaceutical field can investigate the potential of the candidate drugs for use in new indications [7].

As knowledge of biological mechanisms advances and biomedical knowledge is well collected, more accurate and precise computational drug repurposing based on well-curated data has become possible [8]. One computational repurposing framework is a network-based approach that can recommend candidate drugs by observing the complex relationships among biological entities such as drugs, genes, and diseases. Their complex and heterogeneous interactions can be represented by topological structures among nodes and edges in a graph [9–11].

**Fig. 1:**
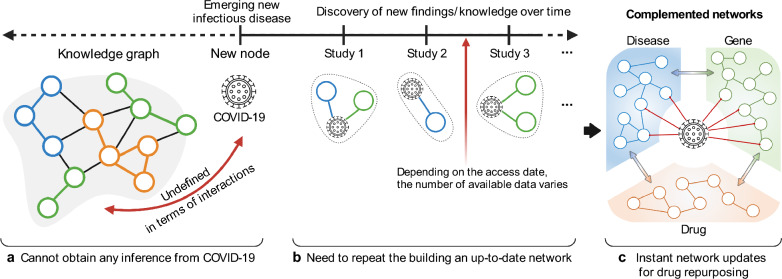
Limitations of network-based approaches in the face of the emerging new infectious disease. **a** New disease (COVID-19) cannot be connected with the knowledge network using data collected before emerging COVID-19. **b** Although the novel associations related to COVID-19 can discover by researchers over time, the final network for drug repurposing can differ depending on the selected dataset. **c** We developed a new method of updating the network instantly with the complementary dataset (discovered findings from studies) to find candidate repurposable drugs for COVID-19

Even though biomedical/pharmaceutical data sources are more readily available than ever, what if a new infectious disease emerges and there is no information about the new disease in the previously accumulated database? This is the scenario the scientific and medical communities faced in the early stages of the COVID-19 pandemic (urgent need for discovering therapeutic treatment for COVID-19). In a network-based approach, the new disease node, i.e. COVID-19, could be introduced in an existing biological network to apply network-based drug repurposing. However, if the new disease is disconnected with the existing network, meaning COVID-19 is undefined in terms of interactions with other components, the disconnected node cannot provide sufficient pharmaceutical evidence or inferences (Fig. 1a). As COVID-19-related research progressed gradually, relational information (such as pathogenesis of COVID-19 and related target genes/proteins) were discovered over time (Fig. 1b). To connect the COVID-19 node to the previously constructed network, the network needs to be rebuilt or updated by incorporating the newly found information. As the information related to COVID-19 discovered, various networks including latest information can be built or updated depending on which association information is used (Fig. 1b). However, it is inefficient to repeat the entire process of building an up-to-date network every time new data sources come in when much new information is revealed and reported from the researchers continuously. Obviously, a more sophisticated network will help predict repurposable drugs with more therapeutic potential [12, 13]. However, in a public health emergency as we have experienced, it is also crucial to provide even potential evidences for candidate drugs to allow pharmaceutical experts to conduct early-stage trials, even if the candidate drugs were predicted from a relatively less elaborate networks. Inspired by this, we propose a network-based method for rapid screening of repurposable drugs that enables to efficiently incorporate the complementary information for a new entity into existing networks (Fig. 1c).

In this study, we assumed that we faced the initial/early stage of the COVID-19 pandemic. We collected the relationship information for COVID-19 reported as of May 2020. Through this paper, we showed the simulation of how to overcome the lack of information related to new infectious diseases and how to validate the candidate repurposable drugs from the proposed complementary network. First, a backbone network without COVID-19 related information was created. The backbone network consisted of heterogeneous components such as diseases, genes, and drugs which connected with each other based on calculated proximity. The backbone network was processed as a multi-layered network that consists of three different single networks including a disease-disease network, a protein–protein interaction network and a drug-drug network. To introduce the novel disease into the constructed backbone network, a network-based complementary linkage method was developed to estimate the auxiliary connections between the new disease node and the heterogeneous multi-layered network. In our previous study, we already developed complementary method for enhancing a single disease network or a single drug network to improve their connectivity, but there was a limitation in the previous method of not being able to make estimating multiple connections at once—the previous method can only connect one edge per iteration, and thus the overall quality of estimated complementary edges is dependent on the order of connected edges [14, 15]. We improved the complementary linkage method to enable estimating a batch of multiple connections at once by applying the enhanced multi-layered network with heterogeneous or homogenous data. Next, with the complemented network complemented by the novel disease node and its estimated connections, repurposable drug were screened via graph-based semi-supervised learning, which propagates label information along with the multi-layered topological structure. The label propagation algorithm can produce a ranked list of prioritized candidate drugs with normalized scores. We then took the candidate drugs with the highest scores and looked for evidence of associations with patient medication orders and COVID-19 related phenotypes using electronic health record information from the Penn Medicine health system.

## Methods

### Overview of proposed network-based drug repurposing method

**Fig. 2:**
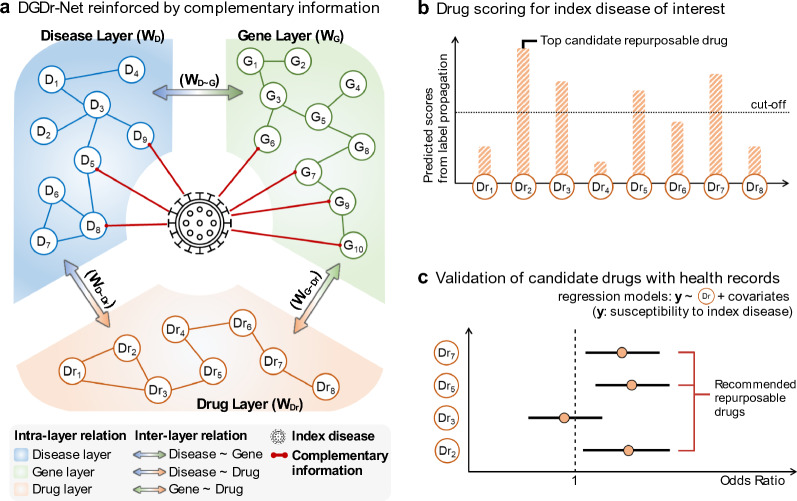
Schematic description of proposed network-based drug repurposing method. **a** Complementing the backbone network with COVID-19 information. **b** Drug scores for COVID-19 by applying graph-based semi-supervised learning. **c** Association analysis with the predicted candidate drugs and electronic health records to evaluate drug scoring

We propose a network-based drug repurposing method for rapid screening to respond to the situation of an emerging new disease. The proposed method can quickly update and augment a knowledge-driven comprehensive multi-layered network with novel disease relationship data and then prioritize the candidate repurposable drugs based on the complemented network. First, we constructed the multi-layered network as a backbone by collecting data from publicly available databases (Additional file 2: Table. S1). As described above, we assumed that COVID-19 emerged as a new disease node that was not connected to the multi-layered backbone network. Additional relational information about COVID-19 to be augmented to the backbone network was collected from papers published or shared on preprint servers (medRxiv and bioRxiv) before May 2020 and contains diseases comorbid with and genes relevant to COVID-19 [16]. A network-based complementary linkage method was developed to estimate connections between the novel COVID-19 disease node and the backbone network (Fig. 2a). The complementary linkage method can determine whether the additional relational information can harm the backbone network when the arbitrary edges provided by users are connected. Then, graph-based semi-supervised learning (SSL) was applied to prioritize the repurposable candidate drugs for COVID-19 (Fig. 2b). The graph-based SSL can predict candidate drugs by leveraging the underlying structure of complemented network when only one label information was given [17–19]. The prioritized list of candidate drugs was validated with electronic health records at Penn Medicine.

### Construction of backbone network

**Fig. 3:**
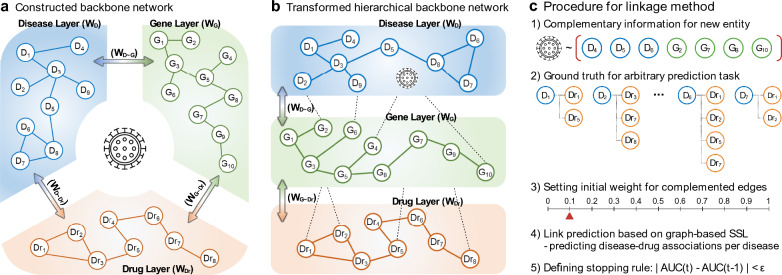
Systematic process of complementary linkage method for multi-layered network

The Disease-Gene-Drug Network was constructed as a backbone network to represent relationships between different biological components. The backbone network is a multi-layered heterogeneous graph,${\varvec{G}}=({\varvec{V}},{\varvec{W}},\boldsymbol{ }{\varvec{S}})$, where the set of nodes ${\varvec{V}}$ represents diseases, genes, and drugs according to the set of layers ${\varvec{S}}=\{D, G, Dr\}$ respectively, and the similarity matrix ${\varvec{W}}$ represents the relationships within and across layers (Fig. 3a). Since the network is multi-layered, we defined intra-layer relations and inter-layer relations by decomposing the similarity matrix ${\varvec{W}}$ as ${{\varvec{W}}}^{\left\{\mathrm{intra}\right\}}$ and ${{\varvec{W}}}^{\left\{\mathrm{inter}\right\}}$. The intra-layer relation depicts a single network such as a disease-disease network, a protein–protein interaction network, or a drug-drug network. The similarity for single network was quantified by calculating the cosine similarity using the respective association vectors. For example, similarity between diseases were calculated by disease-gene association vectors. The inter-layer relation represents the connections between different single networks (different layers), which consist of disease-gene association, disease-drug association, and drug-gene associations [20]. More details about constructing network are described in Additional file 1: Extended method, and Additional file 2: Table S1.

### Network-wise complementary linkage method for multi-layered network

The network-based complementary linkage method can estimate connections between a single disease node of COVID-19 and the backbone network. The strategies of the proposed linkage method are as follows: (a) adding initial information provided by users for estimating auxiliary connections (estimated edges), (b) prediction tasks are defined within the backbone network to learn how to estimate new edges between backbone network and novel node, (c) the properties of backbone network are defined as a loss function, and (d) user-provided auxiliary connections are allowed in a complementary process, provided it does not compromise the pre-defined properties of the backbone network (stopping rule).

The proposed steps were used to augment the novel COVID-19 node into the backbone network. (a) 18 comorbid diseases and 17 related genes were collected from the literature and used initial information (Additional file 2: Table S2). (b) The prediction task was defined as predicting disease-drug associations in the backbone network. The original tripartite multi-layered backbone network was transformed into the hierarchical layered network to facilitate these disease-drug association predictions, ordered by disease, gene, and drug layer (Fig. 3b). When transforming into a hierarchical network, connections between disease and drug layers were deleted and used as the ground truth for the prediction task during complementary process. Notably, we predict the list of drugs when a single disease node is given as a label. The number of iterations is the same as the number of nodes in the disease-disease network. (c) The measuring property of the transformed backbone network was defined as the area under the receiver operating characteristic (AUC) for an index disease of interest. (d) 35 user-provided auxiliary connections were allowed when the average AUC was not decreased. The connection strength increased the search range by 0.1 units (Fig. 3c).

### Drug scoring algorithm with complemented network

A graph-based SSL was employed to prioritize candidate repurposable drugs with the complemented network. Semi-supervised approaches can be used even if the label information is insufficient compared to the conventional supervised approaches that always require a lot of label information [19]. The scoring algorithm propagates the given label information to the underlying structure of the complemented network. It is a more suitable case for employing graph-based SSL when a new disease such as COVID-19 in the previously assumed situation has no therapeutic agent.

The formulation of the scoring algorithm is as follows. Consider we have $m$ diseases, $n$ proteins, and $k$ drugs in complemented network,${\varvec{G}}=({\varvec{V}},{\varvec{W}})$, with set of nodes ${\varvec{V}}(={{\varvec{V}}}_{D}\cup {{\varvec{V}}}_{\mathrm{G}}\cup {{\varvec{V}}}_{\mathrm{Dr}})$ corresponding to the $\left|{\varvec{V}}\right|(=m+n+k)$ nodes. Let ${\varvec{y}}={\left({y}_{1}, \dots , {y}_{\left|{\varvec{V}}\right|}\right)}^{\mathbf{T}}$ denote the initial label set of nodes, and ${{\varvec{f}}={\left[{{\varvec{f}}}^{D},\boldsymbol{ }{\boldsymbol{ }{\varvec{f}}}^{G},\boldsymbol{ }{\boldsymbol{ }{\varvec{f}}}^{Dr}\right]}^{\mathrm{T}}=\left({f}_{1}^{\mathrm{D}}, \dots , {f}_{m}^{\mathrm{D}},{f}_{1}^{\mathrm{G}},\dots ,{f}_{n}^{\mathrm{G}},{f}_{1}^{\mathrm{Dr}},\dots ,{f}_{k}^{\mathrm{Dr}}\right)}^{\mathrm{T}}$ denote the set of resulting scores. Unlike the general classification problem where the target variable has a binary label (‘ + 1’ or ‘-1’), the problem setting of scoring in a semi-supervised approach has a unary label (‘ + 1’) only. More specifically, a disease node of COVID-19 (${v}_{\mathrm{COVID}}^{D}$) is set to a unary label ${y}_{\mathrm{COVID}}\in \left\{+1\right\},$ and the other nodes set to zero (${\varvec{y}}\backslash {y}_{\mathrm{COVID}}\in \{0\})$. In graph-based SSL, there are two assumptions: (a) a loss function that predicted scores in unlabeled nodes should be close to the given label of ${y}_{i}$ in labeled nodes and (b) a smoothness condition that predicted scores in adjacent unlabeled nodes should be close to each other. These assumptions are reflected by the quadratic objective function in Eq. (1) where the graph Laplacian ${\varvec{L}}$ is defined as ${\varvec{L}}={\varvec{D}}-{\varvec{W}}$, ${\varvec{D}}=\mathrm{diag}\left({\sum }_{j}{w}_{ij}\right)$ is diagonal degree matrix of${\varvec{W}}$, and the user-specified parameter $\mu$ trades off loss and smoothness (to reduce computational complexity, $\mu$ is set to $1/{\Vert L\Vert }_{1}$ in this study).

1 $$\mathbf{min}\,{\left({\varvec{f}}-{\varvec{y}}\right)}^{\mathbf{T}}\left({\varvec{f}}-{\varvec{y}}\right)+\mu {{\varvec{f}}}^{\mathbf{T}}L{\varvec{f}}$$

By minimizing objective function in Eq. (1), the closed-form solution becomes${\varvec{f}}={\left({\varvec{I}}+\frac{{\varvec{L}}}{{\Vert {\varvec{L}}\Vert }_{1}}\right)}^{-1}{\varvec{y}}$. The predicted score ${\varvec{f}}$ is produced for all three single layers, but since we are only interested in repurposable drugs in the drug layer, the value of ${{\varvec{f}}}^{Dr}$ is transformed to range from 0 to 1 as ${\widehat{{\varvec{f}}}}^{Dr}=\frac{{{\varvec{f}}}^{Dr}-\mathrm{min}({{\varvec{f}}}^{Dr})}{\mathrm{max}\left({{\varvec{f}}}^{Dr}\right)-\mathrm{min}({{\varvec{f}}}^{Dr})}$. All drugs are sorted in descending order according to the transformed scores ${\widehat{{\varvec{f}}}}^{Dr}$ to prioritize the repurposable drugs for the index disease of interest [15, 17].

### Validation of prediction results with candidate repurposable drugs

The predicted results from the scoring algorithm can provide the prioritized scores of each drug, but it is hard to validate the potential of each candidate drug since there is no pharmacological evidence related to the novel disease COVID-19. Although the listed candidate drugs for the COVID-19 have already been approved to treat other illnesses, it is unreasonable to conclude predict these drugs’ efficacy to treat COVID-19 as these drugs have not yet undergone clinical trials for the COVID-19. However, during global health emergencies such as the COVID-19 pandemic, there is not enough time for clinical trials so an effective indirect verification method is needed. Therefore, candidate drugs were statistically validated using electronic health record (EHR) data.

First, we extracted the clinical records from the Penn Medicine COVID-19 Registry and assigned case/control phenotypes for COVID-19 susceptibility, hospitalization, and severity based on the COVID-19 Host Genetics Initiative phenotype definitions [21]. Then, we built logistic regression models for the effects of the candidate medications from the complemented network on phenotypes (${\varvec{y}}$) related to the index disease using each medication (${\widehat{{\varvec{f}}}}^{Dr}$**)** as a predictor and adjusting for age, gender, and self-reported race as covariates: ${{\varvec{y}}}_{\mathrm{phenotype}} \sim {\widehat{{\varvec{f}}}}_{[i]}^{Dr}+\mathrm{age}+\mathrm{gender}+\mathrm{race}$, where phenotype was one of the EHR-derived COVID-19 phenotypes (i.e., susceptibility, hospitalization, severity, and mortality), and ${\widehat{{\varvec{f}}}}_{[i]}^{Dr}$ is the $\left[i\right]$ ^th^ ranked candidate drug obtained from the complemented network.

The full study protocol for Penn Medicine EHR analysis was approved by the University of Pennsylvania Institutional Review Board (IRB) under the protocol for the study titled “Clinical, social, and genetic risk stratification for COVID-19 outcomes” (Protocol #844,360).

## Results

### Complementing backbone Disease-Gene-Drug network with COVID-19 information

The backbone network was constructed with 591 diseases, 26,681 genes (proteins), and 2,173 drugs by collecting a list of components and relational data from public databases including the Comparative Toxicogenomics Database (CTD), the Search Tool for the Retrieval of Interacting Genes/Proteins (STRING), and DrugBank [22–24]. The backbone disease-gene-drug network had three different single networks: disease-disease network, protein–protein interaction network, and drug-drug network. These single networks were then connected based on the relational data. Intra-layer relationships contained 22,855 disease-disease associations among 591 diseases, 841,068 interactions among 26,681 proteins, and 577,040 drug-drug associations among 2,173 drugs. There were 31,991 disease-gene associations, 76,889 disease-drug associations, 9,540 drug-gene associations in inter-layer relationships.

**Fig. 4:**
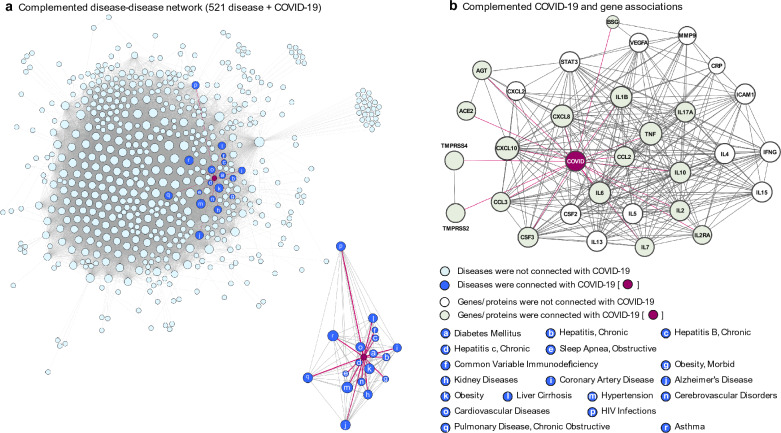
Complemented network with COVID-19 information: **a** complemented disease-disease network, **b** complemented protein–protein interaction network with COVID-19 disease node

In order to incorporate the COVID-19 disease node into the backbone network, we collected initial information for auxiliary connections from the literatures: 18 diseases had been reported as comorbid with COVID-19 (e.g., diseases related to chronic hepatitis, HIV infections, obstructive sleep apnea, hypertension, obesity, diabetes mellitus, kidney diseases, common variable immunodeficiency, liver cirrhosis, coronary artery disease, chronic obstructive pulmonary disease, Alzheimer’s disease, asthma, cardiovascular disease, and cerebrovascular disorders) [16, 25, 26]. Also, we found 17 related genes for a biomarker of therapeutic evidence reported as of May 2020 (e.g., CCL2, TNF, IL10, CXCL8, IL6, IL1B, AGT, IL2, CXCL10, CCL3, TMPRSS2, IL7, IL2RA, CSF3, TMPRSS4, ACE2, and BSG). The connection strength (edge weights) for complementing edges was initially taken at a constant value of 0.1, and 0.1 units increased until the overall AUC decreased. In this analysis, the 35 complemented connections between COVID-19 and other nodes were estimated. Finally, the complemented disease disease-gene-drug network had 1,440,998 (= overall edges in backbone network + 35 complemented edges) associations among 29,446 nodes. Figure 4 shows the complemented disease-gene-drug network with COVID-19. For easier visualization, we decomposed the entire network: the complemented disease-disease network (Fig. 3a) and the subset of the protein–protein interaction network (Fig. 4b). Note that there are no actual and complemented connections between COVID-19 and any drugs due to the defined problematic situations in this study.

### Internal quality check of the complemented network

**Fig. 5:**
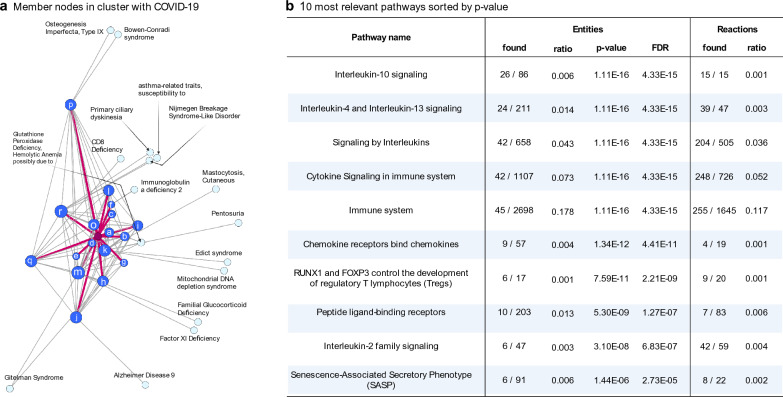
Internal quality check for complemented network: **a** Sub disease-disease network in community with COVID-19, **b** Top 10 significantly enriched pathways with selected 30 gene sets from scoring results

Before applying the drug scoring algorithm to prioritize candidate repurposable drugs, we conducted internal validation of the estimated connection between COVID-19 and other diseases. Although the reliability of the complemented network was verified by complementary process, we performed further quality check of connectivity between COVID-19 and other diseases/genes. To validate the connections focusing on COVID-19, a connectivity check within a single network was performed. First, to detect the community connected with COVID-19 node, the Louvain method for community detection was applied in the complemented single disease-disease network [27]. 33 diseases were belonging to same cluster with COVID-19. Notably, 18 directly- connected comorbidities (used for seed initial information) were included and the remaining 15 diseases were two-hop neighbors with COVID-19 in the cluster (Fig. 5a). Next, quality check for multi-layered network was performed. Scoring algorithms was applied to disease-gene complemented network for predicting COVID-19 related genes. Similar to method in drug scoring, the initial label was set to ${y}_{\mathrm{COVID}}\in \left\{+1\right\}$ and the others were set to {0} and applied graph-based SSL. We substituted the gene scores ${{\varvec{f}}}^{G}$ from the entire predicted results ${\varvec{f}}={\left[{{\varvec{f}}}^{D},\boldsymbol{ }{\boldsymbol{ }{\varvec{f}}}^{G}\right]}^{\mathrm{T}}$. The value of ${{\varvec{f}}}^{G}$ is transformed to range from 0 to 1 via ${\widehat{{\varvec{f}}}}^{G}=\frac{{{\varvec{f}}}^{G}-\mathrm{min}({{\varvec{f}}}^{G})}{\mathrm{max}\left({{\varvec{f}}}^{G}\right)-\mathrm{min}({{\varvec{f}}}^{G})}$. All genes are sorted in descending order according to the transformed scores ${\widehat{{\varvec{f}}}}^{G}$. The overall gene scores were reported in Additional file 3. The top 30 of 26,681 genes sorted by gene score were TMPRSS4, TMPRSS2, ACE2, BSG, IL7, CSF3, CCL3, IL2RA, IL2, IL10, CXCL10, CCL2, IL1B, AGT, CXCL8, IL6, TNF, IFNG, VEGFA, IL4, ICAM1, STAT3, IL13, IL5, IL17A, CSF2, CRP, IL15, CXCL2, and MMP9 in descending order. The pathway enrichment tests were performed with selected gene sets by using an over-representation analysis approach [28]. The 10 most relevant pathways were sorted by p-value (Fig. 5b). The most significant enriched pathways were related to anti-inflammatory phenotypes and/or the human immune system.

### Drug scoring results for prioritizing repurposable drugs

We performed a scoring algorithm to predict candidate repurposable drugs for COVID-19 with the complemented disease-gene-drug network. The initial label of graph-based SSL was set to COVID-19 only (${y}_{\mathrm{COVID}}\in \left\{+1\right\}$). COVID-19 was connected with 18 diseases and 17 genes directly after complementation, but none of the drugs were directly connected with COVID-19. Even though COVID-19 was not directly connected with any drugs, the candidate drugs can be predicted indirectly by considering the proximities between intra-layer relations and inter-layer relations. The scoring algorithms propagates the one positive label from COVID-19 node to remaining unlabeled node with the underlying structure of network. Since there were no approved therapeutic treatments for COVID-19 as of May 2020, there are no ground truths for the predicted results making it difficult to evaluate their accuracy in this study.

**Fig. 6:**
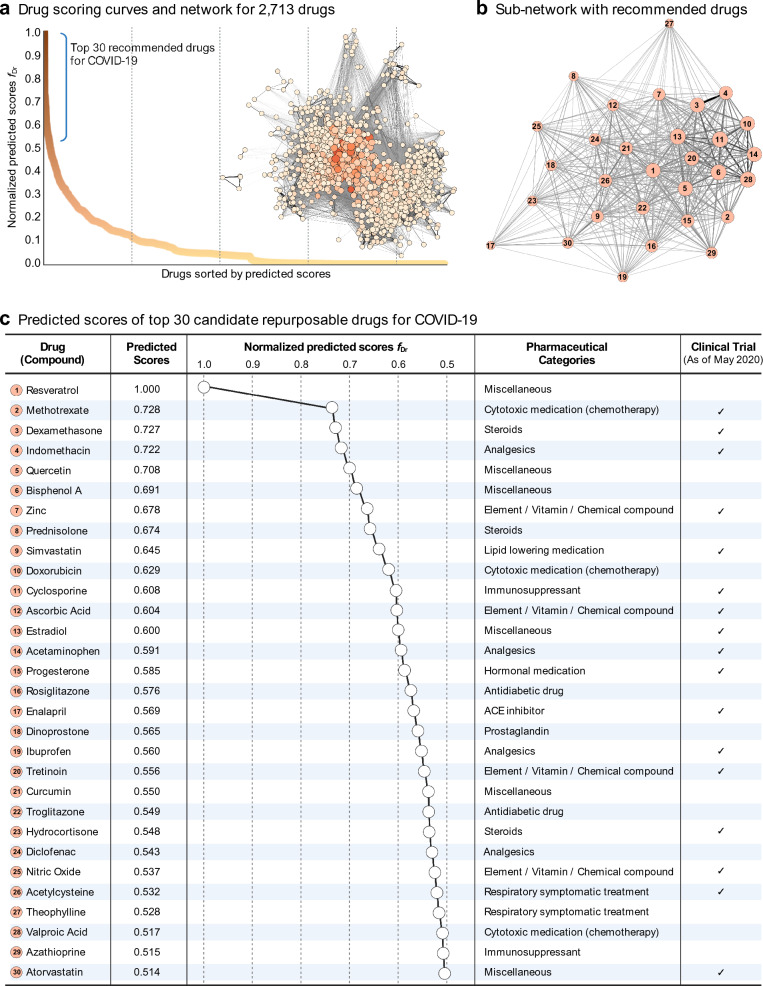
Candidate repurposable drugs with drug scoring curves

The entirety of the drugs as shown in the scoring curves could be candidates for repurposable drugs for COVID-19 (Fig. 6a). The dark to light colors in scoring curves and networks’ nodes represent the normalized scores. In order to provide a list of candidate drugs with data-driven evidence, we recommended the top-30 candidate repurposable drugs from the scoring curve (normalized scores > 0.5). Figure 6b depicts the sub-network with the recommended candidates and Fig. 6c shows the detailed predicted scores. Steroids such as dexamethasone, prednisolone, and hydrocortisone were recommended as top candidates. Among them, dexamethasone, an anti-inflammatory drug, had the highest scores. Dexamethasone is a low cost steroid that reduces inflammation by mimicking anti-inflammatory hormones produced by the body. COVID-19 treatment guidelines recommend using 6 mg per day dose of dexamethasone for up to 10 days for hospitalized patients with COVID-19.

In addition, we were able to find 17 cases preparing study protocols or recruiting for clinical trials with recommended drugs as of the end of May 2020 (regardless of whether or not they were discontinued as of September 2022, reported in https://clinicaltrials.gov). In addition, we searched the literature for evidence or possible relationships between the list of drugs and COVID-19 in order to investigate the potential of repositioning in the recent studies. Since the 30 candidate repurposable drugs were prioritized in the complemented network based on past time study points. Relevant studies identified for determining possible therapeutic candidate in COVID-19 can be found in the additent. (Additional file 2: Table S2).

### Associations of prioritized repurposable drugs with COVID-19 phenotypes using electronic health records

To provide additional evidence for the utility of network-based drug repurposing with complemented network, we conducted an EHR-based medication analysis using the prioritized candidate drugs and several COVID-19 phenotypes. Among ~ 160 K patients in the Penn Medicine COVID-19 registry, which includes all COVID-19 RT-PCR test results within the health system, as of October 2021, we extracted medication order data to investigate associations with the candidate drugs and various COVID-19 outcome phenotypes. To perform these statistical analyses, we assigned case and control status for each of three COVID-19 outcomes: (a) COVID-19 susceptibility, (b) COVID-19 positive hospital admission, and (c) COVID-19 severity.

(a) COVID-19 susceptibility was determined by a positive RT-PCR test whereas patients who had only ever had a COVID-19 negative RT-PCR test in the registry were labeled as a control. (b) A COVID-19 positive hospital admission was determined by an inpatient hospitalization with a primary diagnosis ICD-10 code U07.1 used for COVID-19 diagnosis and a positive RT-PCR test or a primary diagnosis of an ICD code indicative of a COVID-19 related symptoms, a positive RT-PCR test, and a clinician chart review for admissions dated prior to the usage of U07.1. Remaining cases from the COVID-19 susceptibility phenotype were considered as controls. (c) COVID-19 severity was determined by use of ventilator and/or an intensive care unit stay during an inpatient hospitalization as defined previously in (b) while remaining cases from the COVID-19 hospitalization phenotype were considered as controls.

We ran logistic regressions for each outcome, using a selected candidate drug as a predictor and adjusting for age, gender, and the self-reported race as described in Methods. We only included patients with four or more encounters within the Penn Medicine hospital system prior to their COVID-19 RT-PCR test to reduce bias, i.e. patients who may have received COVID-19 related care at Penn Medicine but were not regular patients and thus would not have historical medication order data.

**Fig. 7:**
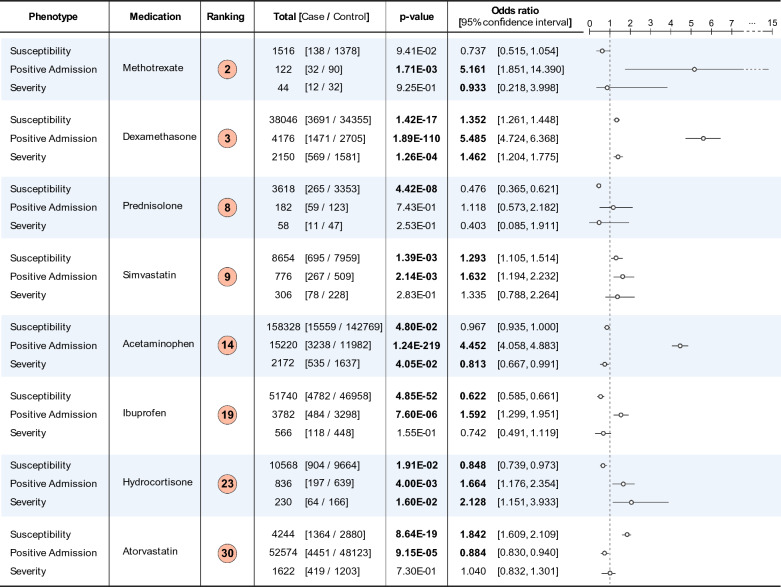
List of statistically significant medications

We were able to conduct this analysis for 23 out of 30 medications using the EHR data. 8 candidate drugs were found to be statistically significantly associated with at least one of the COVID-19 phenotypes (Fig. 7, Bonferroni p-value < 0.05). The most significant association was between a COVID-19 positive hospital admission and the NSAID acetaminophen (p-value < 1e-100, OR = 4.5, 95% CI = [4.1, 4.9]). As acetaminophen is a commonly taken drug, the higher odds of hospital admission could be confounded by sicker patients taking acetaminophen for symptoms of COVID-19 at higher rates prior to their hospital admissions. In contrast, though not significant at the Bonferroni threshold, the association between acetaminophen and the COVID-19 severity phenotype showed a negative direction of effect (OR = 0.81, 95% CI = [0.66, 0.99], unadjusted p-value = 0.04). Ibuprofen, another NSAID, showed a similar trend with an increased odds of a positive hospital admission (OR = 1.6, 95% CI = [1.3, 2.0], p-value = 7.6e-06), but decreased odds of having the COVID-19 susceptibility (OR = 0.62, 95% CI = [0.58, 0.66], p-value = 4.9e-52) and severity (0.74, 95% CI = [0.49, 1.12], p-value = 0.15) phenotypes, though the latter was not significantly different from 1. Several other drugs associated with reduced odds of the COVID-19 susceptibility phenotype included the steroid analogue prednisolone and the lipid lowering medication atorvastatin.

## Discussion

As the current COVID-19 pandemic transitions into an endemic, it is necessary to reflect on the lessons learned to prepare for emerging pandemic risks that may arise in the future. Most significant findings will come from well-designed data and analyses, but at a time when information is insufficient, such as in the early stages of the COVID-19 pandemic, it is also very important to quickly provide information that can help in a public health emergency.

Network-based drug repurposing can provide candidate drugs by comprehensively leveraging complex connectivity among heterogeneous biological entities. However, in the case of a situation where there is no information about the new disease in the previously accumulated database when a new infectious disease emerges, it is challenging to obtain inference until a new disease node like COVID-19 has a connection with other components from network-based approaches. To solve this connectivity problem, we developed the network-based complementary linkage method to overcome the deficiency of disconnections between new emerging diseases and an already-constructed network. One of the objectives of this study is to quickly provide medical or pharmaceutical experts with a curated catalog of potentially repurposable drugs, serving as a prompt for investigating their potential during a public health emergency. It is unreasonable to immediately use the results of computational drug repurposing as alternatives to existing therapeutics without any clinical verification, but the provision of prioritized candidate drugs based on evidence of knowledge can reduce the number of failures in early-stage trials.

From this point of view, it is more important to provide evidence for utilization through medication association analysis with EHR, rather than simply providing a list of candidates. However, the EHR analysis highlighted several challenges of searching for associations between medication use and clinical outcomes in observational medical records data. First, it is difficult to determine a direct cause of a particular medication towards an outcome; for example, a drug could show a strong association with a negative disease outcome simply because it is more frequently prescribed to patients with severe disease and not due to a negative effect of the drug itself. Similarly, patients can, and often are, taking multiple drugs simultaneously making it difficult to control for potential drug interaction effects. Lastly, using medication orders data relies on the assumption that patients filled the order and took the medication as prescribed. This assumption has more potential impact on inferences that require some knowledge of the patient outside of the healthcare system, such as for the COVID-19 susceptibility phenotype which looks prior to the patient’s positive test, and is less of a concern for hospital admission outcomes where detailed, structured electronic health record data provide more certainty of the timing and administration of the drug. Despite these limitations, EHR remains an invaluable resource for identifying potential candidates for drug repositioning, particularly in the context of emerging new disease, and this method could further be expanded upon allowing for more sophisticated emulated clinical trials in large diverse patient populations.

Several prioritized drugs may be considered for the management of severe COVID-19 symptoms. First, dexamethasone, prednisolone, and hydrocortisone are steroid analogues, which affect immune and inflammatory functions. There have been reports suggesting that steroids may be effective in the control of systemic inflammation or ‘cytokine storm’ in severe COVID-19 cases [29], and there are several on-going trials on the effectiveness of steroid treatment [30]. The current study also supports the possibility of steroid therapy in patients with COVID-19. Until now, there has been controversy regarding the use of NSAIDs in COVID-19 patients. With the inclusion of acetaminophen as one of the highlighted drugs in our study, we wish to bring attention to the role NSAIDs may play in helping an individual with COVID-19. Given the body’s inflammatory response to the virus, researchers have been studying the effects of some immune-modulating drugs including methotrexate and cyclosporine [31, 32], although there is a paucity of information on other immune-modulating medications or cytotoxic drugs including azathioprine, doxorubicin, valproic acid, and arsenic trioxide.

Based on the current study, further studies are needed to evaluate the possibility of immune-modulating drugs in the context of COVID-19. Several drugs also require attention, such as ACE inhibitors (enalapril), lipid lowering medications (simvastatin), hormonal medications (estradiol, progesterone) and antidiabetic drugs (rosiglitazone, troglitazone). These medications may be more effective in populations with specific comorbidities such as kidney disease, diabetes, or coronary/cardiovascular disease there is need to evaluate the efficacy of these medications in these populations. However, of course, before any of these potential treatments are given to help patients suffering from COVID-19, rigorous clinical trials are required.

## Conclusion

In this study, we developed a network-based drug repurposing method for rapid screening to respond to the situation of a new emerging disease, to present solutions and new methodologies to address the lessons learned from the COVID-19 pandemic in terms of network-based drug repurposing. We proposed the network-based complementary linkage method to overcome the deficiency of disconnections between new emerging diseases and an already-constructed network. To simulate and test our proposed method, we assumed situation as an early stage of the pandemic with insufficient information related to COVID-19. We constructed a backbone network using publicly available biomedical and pharmaceutical data and fragmented COVID-19-related information was reinforced into the backbone network by applying the proposed complementary linkage method. To translate the complemented network for finding candidate treatments, network-based label propagations were applied and we validated the prioritized candidate drugs with EHR-based medication analysis.

There are several limitations of our studies. From the point of view of the biomedical networks, this study only investigated the heterogeneous relationships between disease, genes and drugs. However, to develop more sophisticated repurposable drugs, complex relationship information can be utilized in the network. For example, single-nucleotide polymorphisms obtained through phenome-wide association studies, or green nanomaterials targeting specific cells or mitochondria can be utilized [33, 34]. Another particular limitation of our study is the relatively small sample size of the few databases we utilized; however, this concern is quickly alleviated as the robust yet flexible nature of a network-based approach allows us to very easily supplement and correct our current model. As we receive the newest information regarding the novel coronavirus, we can easily update the candidate drug/gene components of our networks, perform a set of updated calculations and generate an updated gene and drug candidate list almost instantly. With this in mind, we hope our approach may help clinicians and scientists make the difficult decisions regarding which drugs or gene targets to test first in this global race for a cure.

## Supplementary Information

**Additional file 1.** Extenede Methods.

**Additional file 2: Table S1.** Data for constructing backbone network. **Table S2. **COVID-19 relational data.

**Additional file 3. **List of gene/protein scores for COVID-19.

## Data Availability

Data for constructing network are publicly available at CTD (the Comparative Toxicogenomics Database, http://ctdbase.org/), STRING (https://string-db.org/), and DrugBank (https://go.drugbank.com/). The Penn Medicine electronic health record data cannot be shared publicly due to the violation of patient privacy and the absence of informed consent for data sharing.
