# Supplementary material for: Development of complemented comprehensive networks for rapid screening of repurposable drugs applicable to new emerging disease outbreaks
Source: J Transl Med. 2023 Jun 26;21:415. doi: 10.1186/s12967-023-04223-2 (PMC10291757; doi:10.1186/s12967-023-04223-2)
Supplement: Supplementary file 1 — Additional file 1. Extenede Methods. [file 12967_2023_4223_MOESM1_ESM.pdf]

Additional File 1: Extended Method

1. Constructing Backbone Disease-Gene-Drug network

In order to observe relationships between diverse components such as diseases, genes and drugs, we construct the Disease-Gene-Drug Networks, which has a tripartite relationships among components in the form of layered network structure. The backbone network is a multi-layered heterogeneous graph, $\boldsymbol{G}_{DGDr} = (\boldsymbol{V}, \boldsymbol{W}, \boldsymbol{S})$, where the set of nodes $\boldsymbol{V}$ represents diseases, genes, and drugs according to the set of layers $\boldsymbol{S} = \{D, G, Dr\}$ respectively, and the similarity matrix $\boldsymbol{W}$ represents the relationships between nodes or layers. Given *m* diseases, *n* genes, and *k* drugs, $\boldsymbol{V}$ is a union set of nodes in three layers and a cardinality of node set is $|\boldsymbol{V}|(= m + n + k)$. The similarity matrix $\boldsymbol{W}_{|V|\times|V|}$ can be expressed in block-wise matrix as Eq. (1) and it can be decomposed into the sum of the diagonal block matrix $\boldsymbol{W}^{\{\text{intra}\}}$) for the intra-layer relations and the off-diagonal block matrix $\boldsymbol{W}^{\{\text{inter}\}}$ for the inter-layer relations.

$$\boldsymbol{W} = \boldsymbol{W}^{\{\text{intra}\}} + \boldsymbol{W}^{\{\text{inter}\}} = \begin{bmatrix} \boldsymbol{W}_{\text{D}} & \boldsymbol{W}_{\text{D}\sim\text{G}} & \boldsymbol{W}_{\text{D}\sim\text{Dr}} \\ \boldsymbol{W}_{\text{D}\sim\text{G}}^{\text{T}} & \boldsymbol{W}_{\text{G}} & \boldsymbol{W}_{\text{G}\sim\text{Dr}} \\ \boldsymbol{W}_{\text{D}\sim\text{Dr}}^{\text{T}} & \boldsymbol{W}_{\text{G}\sim\text{Dr}}^{\text{T}} & \boldsymbol{W}_{\text{Dr}} \end{bmatrix} \quad (1)$$

The intra-layer relation represents a similarity within homogeneous components with respect to disease ($\boldsymbol{W}_{\text{D}}$), gene ($\boldsymbol{W}_{\text{G}}$), drug ($\boldsymbol{W}_{\text{Dr}}$) layers respectively. The inter-layer relation represents the connections between heterogeneous layers (e.g., $\text{W}_{\text{D}\sim\text{G}}$ is connections between disease and gene layers). The following sections introduce the constructions of intra- and inter-layers.

Single network construction (intra-layer relation) According to the set of layers $\boldsymbol{S} = \{\text{D}, \text{G}, \text{Dr}\}$ in the backbone network, three different single networks were constructed as the disease-disease network ($\boldsymbol{G}_D$), gene-gene network ($\boldsymbol{G}_G$) and drug-drug network ($\boldsymbol{G}_{Dr}$). The disease-disease network is an undirected and weighted graph $\boldsymbol{G}_D = (\boldsymbol{V}_D, \boldsymbol{W}_D)$. To calculate similarity matrix $\boldsymbol{W}_D$, we generated the disease-gene association matrix $\boldsymbol{R}_{D\sim G(m\times n)}$ for *m* diseases and *n* genes obtained from the CTD database. The entry of this matrix $\boldsymbol{R}_{D\sim G[i,j]}$ is 1 if disease *i* is associated with gene *j* and 0 otherwise (Note that attribute value 0 in disease vector does not mean that disease has no relationship with a certain gene. It could occur that the association has not been found yet). Then, each disease vector had an *n*-dimensional gene vector. Similarity between disease *i* and *j* was calculated by cosine similarity, $w_{ij} = (v_i \cdot v_j)/(\|v_i\| \cdot \|v_j\|)$.

For gene-gene network $\boldsymbol{G}_G = (\boldsymbol{V}_G, \boldsymbol{W}_G)$, the protein-protein interactions (PPIs) were extracted from the STRING database. To avoid false positive information, *n* genes (proteins) and their interactions were selected with a high confidence level (≥ 0.7). The edge stands for protein interaction, in which the edge weight indicates the presence or absence ('1' or '0' respectively).

In a similar manner to disease-disease network, the drug-drug network $\boldsymbol{G}_{Dr} = (\boldsymbol{V}_{Dr}, \boldsymbol{W}_{Dr})$ was constructed using drug-target gene association matrix $\boldsymbol{R}_{Dr\sim G(k\times n)}$ for k drugs and n target-genes from DrugBank and CTD database. The similarity matrix for the drug-drug network $\boldsymbol{W}_{\mathrm{Dr}}$ was calculated by cosine similarity.

Connection of multiple single networks (inter-layer relation) Each single network was connected by the relational data obtained from CTD and DrugBank. The off-diagonal block matrix in Eq. (1) represents the connection strengths between components in different layers. The connection weights can be taken binary values of 1 if there are associations between two nodes in different layers and 0 otherwise: disease i in $\boldsymbol{V}_{\mathrm{D}}$ and drug j in $\boldsymbol{V}_{\mathrm{Dr}}$ were connected in DGDr-Net if drug j was used as a treatment for disease i. Notably, the backbone network had a tripartite relationship between three different single networks. Edges for intra-layer relations represent associations between components in the same layer. Edges for inter-layer relations represent associations between components in different layers. By applying graph-based machine learning and observing their tripartite relationships, we could easily find and screen the repurposable drugs for index disease of interest.
