# Supplementary material for: Development of complemented comprehensive networks for rapid screening of repurposable drugs applicable to new emerging disease outbreaks
Source: J Transl Med. 2023 Jun 26;21:415. doi: 10.1186/s12967-023-04223-2 (PMC10291757; doi:10.1186/s12967-023-04223-2)
Supplement: Supplementary file 2 — Additional file 2: Table S1. Data for constructing backbone network. Table S2. COVID-19 relational data. [file 12967_2023_4223_MOESM2_ESM.pdf]

Additional File 2: Table S1 and Table S2

Table S1. Data for constructing backbone network

**Description**		**# of data**	**Data sources**	**Released date**
**Components**	Diseases	591	CTD, MeSH descriptor	2020-03-29
	Genes (Proteins)	26,681	STRING	2018-12-20
	Drugs (Compounds)	2,173	DrugBank, CTD	2020-04-22
**Relational data**	Disease-Gene associations	31,991	CTD	
	Disease-Drug associations	76,889	DrugBank, CTD	
	Protein-Protein Interactions	841,068	STRING	
	Drug-Gene associations	9,540	DrugBank, CTD	

Table S2. COVID-19 relational data

**Relational Data**	**Diseases**	**Sources**
18 Comorbidity Diseases	Chronic hepatitis, B, C	Reference [S1-S6] (Published/Preprint before June 2020)
	HIV infections	
	Obstructive sleep apnea	
	Hypertension	
	Obesity	
	Diabetes mellitus	
	Kidney diseases	
	Common variable immunodeficiency	
	Liver cirrhosis	
	Coronary artery disease	
	Chronic obstructive pulmonary disease	
	Alzheimer's disease	
	Asthma	
	Cardiovascular disease	
	Cerebrovascular disorders	
Related genes	CCL2, TNF, IL10, CXCL8, IL6, IL1B, AGT, IL2, CXCL10, CCL3, TMPRSS2, IL7, IL2RA, CSF3, TMPRSS4, ACE2, and BSG	* CTD (reported before June 2020)

[S1] Guan W-j, Liang W-h, Zhao Y, Liang H-r, Chen Z-s, Li Y-m, Liu X-q, Chen R-c, Tang C-l, Wang T: Comorbidity and its impact on 1590 patients with Covid-19 in China: A Nationwide Analysis. European Respiratory Journal 2020, 55.
[S2] Richardson S, Hirsch JS, Narasimhan M, Crawford JM, McGinn T, Davidson KW, Barnaby DP, Becker LB, Chelico JD, Cohen SL: Presenting characteristics, comorbidities, and outcomes among 5700 patients hospitalized with COVID-19 in the New York City area. Jama 2020.
[S3] Li B, Yang J, Zhao F, Zhi L, Wang X, Liu L, Bi Z, Zhao Y: Prevalence and impact of cardiovascular metabolic diseases on COVID-19 in China. Clinical Research in Cardiology 2020, 109:531-8.
[S4] Wang B, Li R, Lu Z, Huang Y: Does comorbidity increase the risk of patients with COVID-19: evidence from meta-analysis. Aging (Albany NY) 2020, 12:6049.
[S5] Cheng Y, Luo R, Wang K, Zhang M, Wang Z, Dong L, Li J, Yao Y, Ge S, Xu G: Kidney disease is associated with in-hospital death of patients with COVID-19. Kidney international 2020.
[S6] Kuo C-L, Pilling LC, Atkins JL, Masoli JA, Delgado J, Kuchel GA, Melzer D: APOE e4 genotype predicts severe COVID-19 in the UK Biobank community cohort. medRxiv 2020.
